# Interacting Agricultural Pests and Their Effect on Crop Yield: Application of a Bayesian Decision Theory Approach to the Joint Management of *Bromus tectorum* and *Cephus cinctus*

**DOI:** 10.1371/journal.pone.0118111

**Published:** 2015-02-18

**Authors:** Ilai N. Keren, Fabian D. Menalled, David K. Weaver, James F. Robison-Cox

**Affiliations:** 1 Washington Department of Fish and Wildlife, Olympia, Washington, United States of America; 2 Department of Land Resources and Environmental Sciences, Montana State University, Bozeman, Montana, United States of America; 3 Department of Land Resources and Environmental Sciences, Montana State University, Bozeman, Montana, United States of America; 4 Department of Mathematical Sciences, Montana State University, Bozeman, Montana, United States of America; Louisiana State University & LSU AgCenter, UNITED STATES

## Abstract

Worldwide, the landscape homogeneity of extensive monocultures that characterizes conventional agriculture has resulted in the development of specialized and interacting multitrophic pest complexes. While integrated pest management emphasizes the need to consider the ecological context where multiple species coexist, management recommendations are often based on single-species tactics. This approach may not provide satisfactory solutions when confronted with the complex interactions occurring between organisms at the same or different trophic levels. Replacement of the single-species management model with more sophisticated, multi-species programs requires an understanding of the direct and indirect interactions occurring between the crop and all categories of pests. We evaluated a modeling framework to make multi-pest management decisions taking into account direct and indirect interactions among species belonging to different trophic levels. We adopted a Bayesian decision theory approach in combination with path analysis to evaluate interactions between *Bromus tectorum* (downy brome, cheatgrass) and *Cephus cinctus* (wheat stem sawfly) in wheat (*Triticum aestivum*) systems. We assessed their joint responses to weed management tactics, seeding rates, and cultivar tolerance to insect stem boring or competition. Our results indicated that *C. cinctus* oviposition behavior varied as a function of *B. tectorum* pressure. Crop responses were more readily explained by the joint effects of management tactics on both categories of pests and their interactions than just by the direct impact of any particular management scheme on yield. In accordance, a *C. cinctus* tolerant variety should be planted at a low seeding rate under high insect pressure. However as *B. tectorum* levels increase, the *C. cinctus* tolerant variety should be replaced by a competitive and drought tolerant cultivar at high seeding rates despite *C. cinctus* infestation. This study exemplifies the necessity of accounting for direct and indirect biological interactions occurring within agroecosystems and propagating this information from the statistical analysis stage to the management stage.

## Introduction

Encouraging positive biotic interactions, while maintaining acceptable levels of production, is implicit in the concept of integrated pest management (IPM hereafter). Yet, while IPM conceptually emphasizes the need to consider the ecological context where multiple organisms coexist, programs are often designed around a single species, usually following a preemptive approach where decision such as cultivar type and seeding rate are made prior to the detection of the pest [1]. This single-pest management approach may not provide satisfactory solutions when confronted with the dynamic and complex interactions occurring between multiple pests at the same or different trophic levels [2], [3]. Replacement of the current single-species pest management paradigm with more sophisticated, biologically-based programs requires an understanding of the direct and indirect interactions occurring between the crop and all categories of pests in the IPM decision-making process [4].

In the Northern Great Plains, the landscape homogeneity and high commodity specialization of conventional agriculture within the native shortgrass prairie ecosystem that dominates this region has resulted in large monocultures of wheat (*Triticum aestivum* L.), an associated specialized pest complex, and an increased reliance on off-farm synthetic inputs. Two key members of this pest complex include *Bromus tectorum* L. (downy brome, cheatgrass) and *Cephus cinctus* Norton (Hymenoptera: Cephidae) (wheat stem sawfly). Despite their ubiquity, interactions, and impacts, no formal attempt has been made to address their joint management. Furthermore, to our knowledge, no attempt has been made to develop a modeling framework that considers multi-pest management decisions while taking into account direct and indirect interactions among species belonging to same or different trophic levels. The *B. tectorum—C. cinctus*—wheat complex represents an ideal case study to assess approaches to integrate direct and indirect multi-trophic interactions into the management decision process.

*Bromus tectorum* is an increasingly problematic weed in range, wildlands, and crop fields across the region [5]. This weed is typically a winter annual graminoid, but also exhibits summer annual life history traits with seedlings capable of emerging through the winter and into spring [6]. With adoption of conservation tillage, enhanced reliance on broadcast nitrogen fertilizer, and increased use of narrow-spectrum herbicides, *B. tectorum* has become a major weed in winter and spring wheat cropping systems [7]. The similar life form of these two species allows growing populations of *B. tectorum* to effectively outcompete wheat for resources such as light, water, and nutrients [8]. Additionally, the selection of herbicide resistant *B. tectorum* biotypes, including cases of multiple resistance, further complicates its management in small grain crop production systems, with yield reductions ranging between 28% and 92%, depending on weed density, time of emergence, distance to crop [9], and disease status [10], [11].

*Cephus cinctus* is one of the most economically important insect pests of wheat [12]. Currently, no effective insecticide-based program exists to manage *C. cinctus* in small grain systems [13]. This species completes one life cycle per year where all immature stages are spent inside protective plant stems and the resulting crop residue, synchronized with the physiological development of the host. Over their 7 day lifespan during an approximately 28–42 day flight period in the spring, individual *C. cinctus* female adults oviposit mostly in the succulent internodes of the growing plants to improve success of larvae feeding on host parenchyma and vascular tissues [14]. In extensive monocultures, host population densities are high and infestation can reach 80% or more of available stems with females exhibiting strong preferences between species and wheat cultivars [15]. As host plants senesce, a single larva moves toward the bottom of the stem and cuts a v-shaped notch at the base and, below this cut, constructs an overwintering chamber to complete obligatory diapause in the crop residue [16]. Herbivory can cause up to 22% reduction in number and size of wheat kernels [17], with greater yield losses possible under concurrent abiotic stress [18]. Stem cutting weakens the tillers, which subsequently lodge, causing irretrievable loss of mature stems, reduced harvest efficiency and yield, increased volunteer wheat pressure, and loss of anchored residue which may result in greater vulnerability to soil erosion and reduced ability to retain moisture.

Wheat, *B. tectorum*, and *C. cinctus* do not occur in isolation. Evidence suggests that *C. cinctus* oviposition patterns and larval development differ between *B. tectorum* and wheat. Specifically, levels of *C. cinctus* infestation in *B. tectorum* can be up to two-fold higher than in wheat. Also, research has demonstrated much greater *C. cinctus* larval mortality in *B. tectorum* than in wheat as the infested host plants mature [19]. While the exact role of *B. tectorum* in determining *C. cinctus* population dynamics and *vice versa* are largely unknown, these competing outcomes indicate that interactions between these pests can play an important role in their joint management.

Energy, environmental, and economic concerns of current management practices motivated recent attempts to reduce reliance on high-input, single-pest species control methods [20]. However, utilizing ecologically sound management strategies that take advantage of the crop attributes for tolerating pests is a complex task due to the dynamic nature of agroecological systems. For example, agronomic traits that increase crop competitiveness including height, disease resistance, and drought hardiness have the ability to decrease *B. tectorum* impacts [21]. Yet, while increasing crop seeding rates has been suggested as a strategy to suppress weed emergence and growth, evidence suggests that intraspecific competition and environmental conditions may offset the usefulness of these tactics [22]. Also, in the absence of an effective conventional insecticide, mitigation of *C. cinctus* damage is best achieved through the use of solid-stem wheat cultivars that negatively impact the insect eggs and neonate larvae, restrict tunneling, and cause additional mortality in later stages of the stem- boring larvae [23]. However, solid-stem varieties may not be as competitive against weeds and concerns exist regarding yield, efficacy, and market opportunities. Furthermore, solid-stem wheat varieties yield variable results as a *C. cinctus* control method, mostly due to inconsistent pith expression which may be affected by abiotic factors as well as intra- and inter-specific competition [24], [25]. Reflecting these difficulties, a recent survey of farmers across the Northern Great Plains indicated that non-chemical pest management practices such as modifying planting densities or choosing resistant wheat varieties were adopted in only 12% and 33%, respectively, of the area planted with spring wheat [26].

Adoption of ecologically-based pest management requires increased understanding of the direct and indirect interactions occurring among pests belonging to the same or different trophic levels. In this paper, we assessed a formal framework for evaluating outcomes of commonly used crop management practices to reflect a total-system approach to pest management that target species at two trophic levels. Specifically, we used a Bayesian decision theoretic approach in combination with path analysis to probabilistically model wheat grain yield while accounting for how crop variety, crop seeding rate, and herbicide application rate jointly affect *B. tectorum* as well as *C. cinctus* abundance and impact. This approach allowed us to jointly incorporate direct and indirect biological interactions occurring within the agroecosystem in a predictive model of wheat yield, rather than just evaluating the individual effect of each pest- or crop-management tactic. To meet our objective, we developed our model based on empirical data of naturally occurring pest levels and wheat yield observed in a field experiment where the crop was grown under different management scenarios.

## Materials and Methods

### Study Site and Experimental Design

This research was conducted on private land on a commercial farm located near Amsterdam, Montana (45° 45′ 29″ N, 111° 19′ 12″ W, 1490 m elevation). No specific permissions were required to conduct this research and did not involve endangered or protected species. Soil type at the area is classified as fine-silty, mixed Typic Haploborolls Amsterdam silt loam series. The typical frost-free period at the site is 98 d with a 30-year average annual precipitation of 500 mm (1981–2010). Annual temperatures for 2008–2010 averaged 12.4 C, 13 C, and 12.6 C with total precipitation for the duration of the growing season of 252 mm, 270.5 mm, and 302.8 mm, respectively.

Each year from 2008 through 2010, we planted spring wheat in three blocks separated by 10 m buffers within a 1 ha area. To represent a variety of management practices, treatments (crop variety, seeding rate, and herbicide rate) were assigned in a randomized split-split plot design with the same treatment combination assigned to the sub-sub plots (7x7 m) in all three years. Whole plot treatment consisted of two wheat cultivars: 1) *C. cinctus* lodging tolerant Choteau (SFLT hereafter): a semidwarf, solid-stem variety with good resistance to lodging caused by *C. cinctus* stem cutting, and 2) drought and competition tolerant McNeal (DT hereafter): a semidwarf, hollow-stem red chaffed variety with minimal lodging resistance and less resilient straw. The first split treatment consisted of seeding rates to target 125, 250, and 500 plants m^-2^, respectively (low, intermediate, and high hereafter). The second split treatment consisted of sulfosulfuron application at either 0.028, 0.014, or 0.007 kg ha^-1^ of active ingredient to correspond to herbicide application rates of 0.8, 0.4 and 0.2 of the label rate, respectively. These in-crop herbicide treatments were applied on May 20, 2008, May 6, 2009, and May 13, 2010 to generate a range of *B. tectorum* abundance. No other grassy weed species was found in the experimental area and broadleaf weeds in the whole experimental site and grassy weeds in the buffers were chemically controlled following standardized procedures.

Plots were planted on April 11, 2008, April 20, 2009, and March 24, 2010 with a Fabro (Swift Current, SK, Canada) no-till drill with 7 double disc openers spaced 25.4 cm apart and a seeding depth of 3.8–5 cm. At planting, we used a starter fertilizer of a 50:50 mix of ammonium phosphate 11–51–0-(0) and potassium sulfate 0–0–52-(18) at 100 kg ha^-1^ for N-P-K-S rate of 5–25–26–9 kg ha^-1^. Broadcast urea 46–0–0 was added May 30, 2008, May 14, 2009, and May 27, 2010 to achieve 150 kg N ha^-1^ across the field. Urea application rates were based on 54 soil samples obtained to a depth of 61 cm, two in each sub-plot, the previous fall.

### *Bromus tectorum*, *Cephus cinctus*, and Wheat Yield Estimates

Approximately one month after planting, three 0.475 m diameter metal rings were anchored to the ground for the duration of the growing season and used for *B. tectorum*, *C. cinctus*, and wheat data collection. Rings were randomly placed in the north half of each sub-sub-plot and were centered between two wheat rows, covering approximately 80 cm of crop rows. Percent cover of *B. tectorum*, visually estimated by the same observer across the duration of the study, and *B. tectorum* height in each ring were measured twice during each growing season: at peak *B. tectorum* biomass (July 20–21, 2010) and at mechanical harvest of each split-split plot. (September 3, 2010). At peak biomass, two additional rings of the same dimensions were randomly placed in each sub-sub plot and used to visually estimate *B. tectorum* cover and height. After these measures, above ground *B. tectorum* biomass within these two rings was manually harvested, oven dried to a constant mass, and weighed. For each ring, *B. tectorum* cylinder volume (area covered x height) and biomass values were used to estimate weed pressure, a unit-less variable standardized on a 0–1 scale as a relative fraction of the maximum biomass x volume observation. Specifically, the linear relationship between *B tectorum* volume and biomass in the two additional rings was used as a calibration curve to derive expected biomass from volume. Expected *Bromus tectorum* biomass was then standardized such that the maximum value does not exceed one. This adjustment maintained the original rank of an observation as determined visually by the trained observer while smoothing over some of the measurement error known to be associated with visual observations of plant cover. At crop harvest, visual percent cover and *B. tectorum* height were again determined before all plant biomass within each ring was uprooted. *Bromus tectorum* was separated from wheat, oven dried to a constant mass, and weighed.

All wheat stems manually harvested within each ring were taken to the laboratory for examination. Lodged wheat heads from stems cut by *C. cinctus* were separated and counted. All remaining intact stems were dissected lengthwise and inspected under magnification for evidence of *C. cinctus* infestation. These intact stems were categorized as 1) Infested—frass, indicative that *C. cinctus* larval boring occurred, but an unknown mortality factor prevented cutting of the stem, 2) Parasitized—evidence that a bivoltine braconid parasitoid had killed a *C. cinctus* larva, leaving either an overwintering cocoon or an emergence hole that the adult parasitoid chewed when exiting the stem, and 3) Uninfested—wheat stems with no evidence of *C. cinctus* cadavers, frass, or parasitoids. For each ring, wheat stems were grouped by category, counted, and their heads threshed to estimate grain yield. To determine *C. cinctus* infestation, twenty green wheat stems were dissected in-field on July 16, 2010; approximately 11 days after the *C. cinctus* adult flight had ended [12]. To further assess *C. cinctus* status at harvest, a 0.4 m^2^ sample area containing only *B. tectorum* was collected on September 3, 2010 in each split-split-plot.

### Statistical Analysis

We conducted a path analysis of the joint impact of wheat variety, wheat seeding rate, and herbicide application rate on *B. tectorum* pressure, *C. cinctus* abundance, and their interactive impact on crop yield. Path analysis is a multiple regression technique that focuses on estimating covariances of multiple non-independent variables. Arranging these variables in a directed, acyclic, graphical model of hypothesized causal relationships, the covariance matrix can be described as a set of structural regression equations where graph nodes are both responses and predictors [27], [28].

To conduct our analysis, we defined two additional concepts. First, the “total effects” of variable X1 on variable Y represents the direct effect combined with indirect effects through any intermediate variables. Second, “relegation”, a variant to the annihilator approach in linear algebra, is defined as replacing a predictor column in a model matrix with residuals found by regressing that column on the other columns of the model matrix, which are not being replaced [29]. For example consider the partitioned linear model (y, X_1_β_1_ + X_2_β_2_, σ^2^Ω), where y is a random variable whose expected value depends on covariates X_1_ and X_2_ and with variance structure σ^2^Ω, and suppose $\text{P}={\text{X}}_{1}{\left({\text{X}}_{1}^{T}{\text{X}}_{1}\right)}^{-1}{\text{X}}_{1}^{T}$ is the hat matrix of X_1_. In this context the relegated model can be written as (y, X_1_β*_1_ + (I_n_-P)X_2_β_2_, σ^2^Ω) where β*_1_ is now the total effects of X_1_on y, both directly and indirectly via X_2_. Keren [30] demonstrated that in the context of path analysis, β*_1_ and β_2_ are unbiased estimates of total effects and can be derived together with their standard errors by relegating intermediate nodes during model fitting in an iterative fashion.

**Fig 1:**
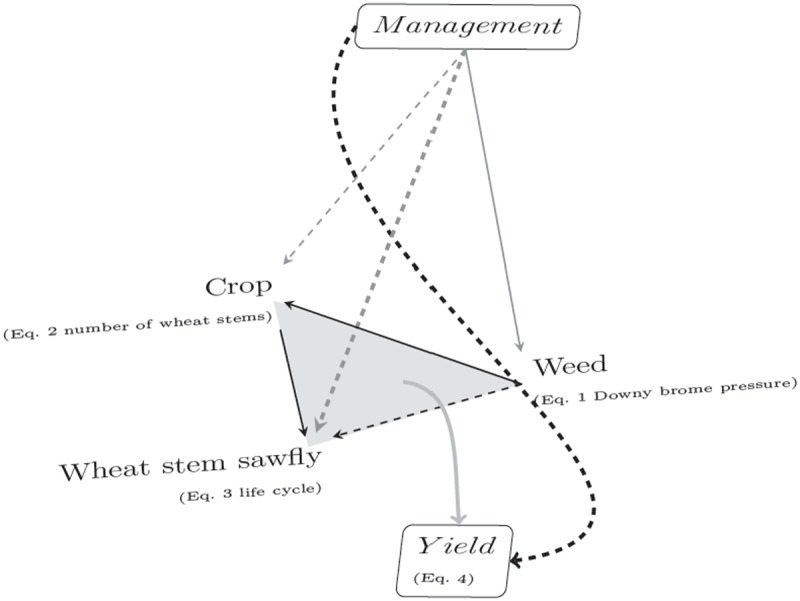
Graphical depiction of the decision model of direct and total effects used to assess the joint effect of *Bromus tectorum*, *Cephus cinctus*, and crop management practices on wheat yield. Equations 1–4 are described in the text. In this model, solid arrows describe direct effects, dashed arrows represent total effects. Aggregation of model components to output is represented as a curved edge, and the gray triangle denotes the collective impact of complex interactions on crop yield.

In our study, the first two years (2008 and 2009) were used to generate pest responses to the applied management practices and to improve our knowledge on the relationships occurring between management decisions, pest abundance, and yield. This, and prior knowledge of *B. tectorum* and *C. cinctus* biology, ecology, and dynamics in wheat-fallow systems [9], [24], [10], [11] was used to construct a structural causal model of hypothesized interactions (Fig. 1), which was tested with data obtained in the 2010 growing season. In our model, interactions were depicted as a set of Generalized Linear Models with appropriate link functions that describe the change in response variables (*Y*_*i*_) by the cumulative impact of *p* management practices combination (***X***_*(ip)*_) and consequential relegated covariate level. *Bromus tectorum* pressure (standardized to a 0–1 scale, see definition above) for ring *i* was modeled as a function of wheat variety, wheat seeding rate, and herbicide application:

$$ln\left(\frac{\mu_{i}}{1-\mu_{i}}\right)=X\beta$$ (1)

where $Y_{i}^{B.\;tectorum}\;\sim \;Beta(\mu_{i}\varphi ,(1-\mu_{i})\varphi )$ with *φ* a dispersion parameter and *μ*_*i*_ the expected value of the Beta distribution such that β is the mean (on the logistic scale) of *B. tectorum* pressure for every wheat variety, wheat seeding rate, and herbicide application rate combination. The two-parameter Beta distribution is a flexible probability density function bounded between 0 and 1 and a natural choice for *B. tectorum* pressure [31]. Number of wheat stems in ring *i* was modeled as a function of both crop management practices and interspecific competition with *B. tectorum*. Therefore:

$$ln(\lambda_{i})=(X+{\hat{\beta }}_{p(i)})\delta \;\;+{\hat{\epsilon }}_{i}\delta_{B.\;tectorum}$$ (2)

where$Y_{i}^{T.\;\;aestivum\;}\;\sim \;Poisson(\lambda_{i})$, **δ** are the total effects of management practices on wheat including any impact of *B. tectorum* pressure, whose level (${\hat{\beta }}_{p(i)}$) is the result of the same management practice decision in ***X***_***i***_ for the given individual observation (*p*(i)). The relegated term ${\hat{\epsilon }}_{i\;}$ contains any additional, individual level information about *B. tectorum* pressure not already explained away by management practices. Therefore δ_*B.tectorum*_ estimates the direct effect of *B. tectorum* pressure on the crop.

*Cephus cinctus* infestation status (counts in each parasite category *k*) in ring *i* was modeled as:

ln(θi kθi[lodged])=(X+δ^p(i))γk+ϵ^⋅i γk[T. aestivum, B. tectorum] (3)

Where $Y_{ik}^{C.\;cinctus\;}\;\sim \;Multinom(\theta_{i},n_{i})$ with *k* being one of “infested”, “parasitized”, “uninfested”, or “lodged stems”, with the last category used as a baseline. Similarly to Equation 2, all consequences of management practices such as herbicide rate, wheat variety, and seeding rate with respect to *B. tectorum* pressure and crop stand complement the direct impact management practices have on *C. cinctus* life cycle in the estimation of ***γ***_***k***_.

Wheat yield per head in ring *i* and *C. cinctus* category *k* was assessed as:

$$\eta_{ik}=(X+{\hat{\gamma }}_{p(i)k})\alpha_{k}+{\hat{\epsilon }}_{\cdot ik}\alpha_{\left[C.\;cintus_{k},\;T.\;aestivum,\;B.tectorum\right]}$$ (4)

Where $Y_{ik}^{yield}\;\sim N(\eta_{ik},\;{\text{Σ}}_{k})$ and *Σ*_*k*_ = *Ωσ_k_* where Ω is the variance structure for our split-split plot design.

Estimated yield per unit area (ring) is computed based on the expected *B. tectorum* pressure (equation 1), number of stems in a ring (equation 2), and consequential *C. cinctus* impact, given by expected yield of wheat heads (equation 4) in the different categories whose frequency is estimated by equation 3. Thus, model predictions for any given combination of management practices account for any expected pest-induced losses associated with that particular combination.

Utilizing a Bayesian approach, we estimated the causal path model parameters and assessed outcomes of management practices with respect to wheat yield in the presence of both *B. tectorum* and *C. cinctus*. Bayesian inference can be described as statistical decision theory in which the process of using empirical evidence to inform a choice between alternative hypotheses or parameter values follows the same logic as a decision making process [32]. For example, Bayesian inference methods allowed us to evaluate and compare choices of management practices because an expected yield can be computed for each field protocol. These were then integrated over the entire joint parameter space, instead of focusing on a single estimated parameter vector. Thus, Bayesian analysis provides a coherent framework for combining all sources of uncertainty in the decision making process, overcomes the problem of discontinuity between the analysis and the managerial decision [33], and fits naturally into our intuitions about that process in the physical world [34].

The joint likelihood of the system described by Fig. 1 is not readily available, but can be estimated by Gibbs sampling (Markov Chain Monte Carlo—MCMC) where the conditional distributions of individual components are fit sequentially and relegated at every iteration. To do this, we used JAGS 3.2.0 [35] via package rjags [36] in R 2.13 [37]. All MCMC simulations consisted of 10 thousand iterations of four chains from overdispersed starting values. After a burnin period of five thousand iterations, two thousand values for every parameter were retained at a thinning interval of 10. Convergence and uni-modality of the distributions for all parameters were assessed visually and were checked for $\hat{R}$ *<* 1.1 [38]. Independence of MCMC draws was confirmed by qualitative comparison of naive standard error to a time-series corrected one [39].

**Fig 2:**
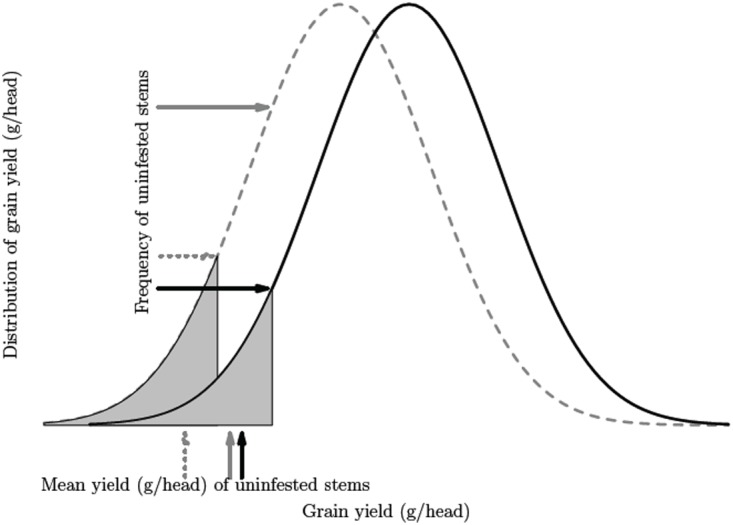
Hypothesized *Cephus cinctus* oviposition choice mechanisms. Standardized grain yield of wheat heads is represented by a N(0, 1) distribution (solid black curve). Dashed gray curve represents wheat which is 25% lower yielding due to either intra- or inter-specific competition and shaded areas represent stems that were not infested by *C. cinctus*. Solid black arrows correspond to observed frequency and mean weight of grain in the uninfested category. Solid gray arrows represent observed value under the same choice mechanism but lower overall yield. Dashed gray arrows represent a modification of the *C. cinctus* choice threshold where wheat stems which could have been considered inappropriate under higher overall potential yields are now selected for oviposition.

In our Bayesian model, the prior distribution had a large variance to reflect a high degree of uncertainty in the estimated values. Thus, the contribution of the prior relative to the likelihood of the data can be considered non-informative in that if the data did not support the prior model specification, no trends will be evident in the posterior. However, the causal or structural model described in Fig. 1 could, in itself, be considered a prior because final yield predictions were conditional on the biological interactions considered which, in part, determine the outcomes. For example, oviposition behavior complicates the characterization of the impact that *C. cinctus* injury has on wheat yield as females prefer larger stem diameter and taller plants, which tend to have larger heads and yields, confounding observed yield reduction estimates [40]. This may be further complicated by *C. cinctus* preference for different hosts such as grassy weeds compared to wheat cultivars [19]. To incorporate these uncertainties, a prior was constructed where we assumed a bell shaped distribution of grain yield for a large unit of wheat stems which the *C. cinctus* female encounters (Fig. 2). For this distribution, there is a minimum value for a stem to be considered suitable for *C. cinctus* oviposition. Two possible oviposition scenarios were evaluated under the condition of reduced wheat yield due to inter- or intra-specific competition (dashed gray curve in Fig. 2). First, *C. cinctus* maintained the same host assessment threshold for a wheat stem to be considered suitable for oviposition (solid gray arrows in Fig. 2), or second, *C. cinctus* responds to the lower overall potential yields by lowering the oviposition threshold (dashed gray arrows in Fig. 2). In the first scenario, we expect to observe more stems below the threshold that are unsuitable. While *C. cinctus* infestation will be reduced under this scenario, wheat yields associated with uninfested stems will not change. Under the second scenario, *C. cinctus* accepts less suitable wheat stems that have lower overall yield potential by modifying the criteria for host selection and subsequently oviposits in these potentially lower yielding stems. Under this latter scenario, there may be less or no significant change in the rate of infestation, but mean yield in the uninfested category would decrease. We used analysis of variance to determine if herbicide rate influenced stem cutting by *C. cinctus* in *B. tectorum* sampled from each split-split-plot at harvest.

## Results

**Fig 3:**
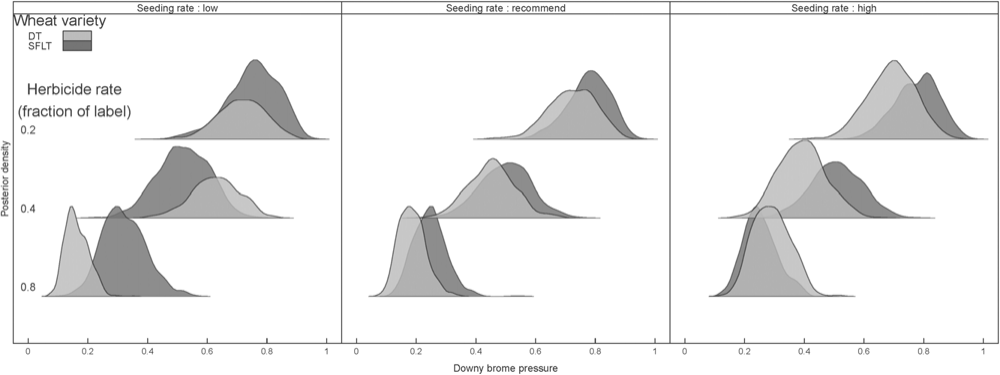
Posterior distributions of *Bromus tectorum* pressure by wheat variety and seeding rate treatments after three years of 0.2x (top), 0.4x (middle), or 0.8x (bottom) herbicide application rate. Crop variety treatments are drought tolerant spring wheat (DT) and *Cephus cinctus* tolerant spring wheat (SFLT).

As expected, herbicide applications modified *B. tectorum* pressure, with values (mean ± 1SD) ranging from 9.1 ± 8.2% pressure at the 0.8x herbicide rate to 64.5 ± 23% pressure at the 0.2x herbicide rate. Increased rates of herbicide also decreased *B. tectorum* pressure in samples (P < 0.05) taken to assess *C. cinctus* survival, but had no effect on the number of *B. tectorum* stems cut by *C. cinctus* (P < 0.05). Stem cutting in *B. tectorum* due to *C. cinctus* ranged from 5.3 ± 5.3 stems m^-2^ at the 0.8x herbicide rate to 3.3 ± 3.6 stems m^-2^ at the 0.4x herbicide rate. Crop seeding rate also affected *B. tectorum* pressure, most noticeably at the 0.4x herbicide rate, with values ranging from 44 ± 24.6% at low seeding rates to 29 ± 20.5% at high seeding rates. Results of the Bayesian decision model on the join impact of herbicide applications, crop seeding rates, and crop variety on *B. tectorum* abundance (Equation 1) confirmed these observations. Specifically, reduced herbicide rates resulted in an increase in *B. tectorum* pressure, with high seeded-DT being an effective management option in suppressing *B. tectorum* particularly in the 0.4x herbicide application rate (Fig. 3).

**Fig 4:**
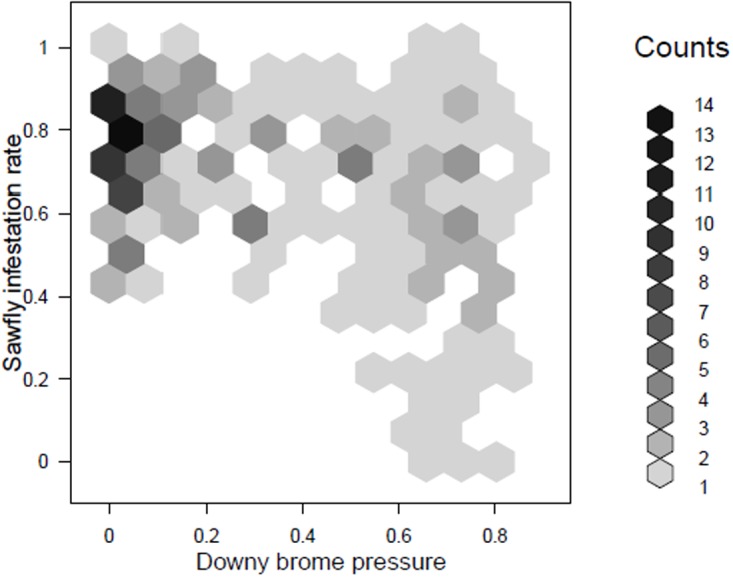
Joint histogram of *Cephus cinctus* infestation rate and *Bromus tectorum* pressure, a unit-less variable calculated from volume (cover x height) and biomass, in spring wheat fields averaged across crop seeding rate and crop variety.

**Fig 5:**
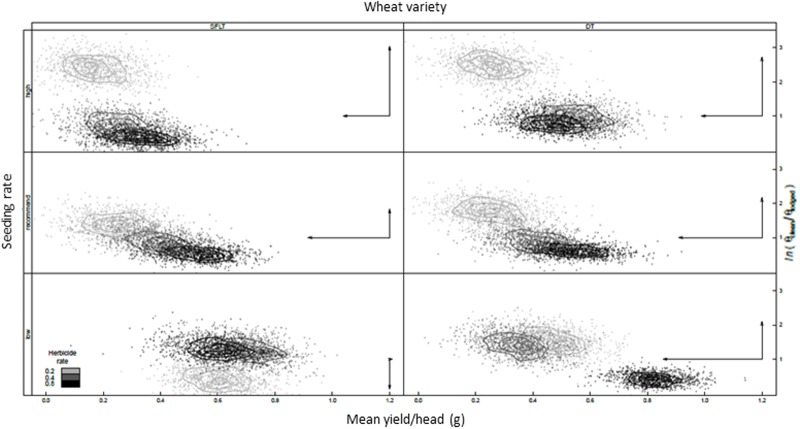
Joint posterior density for the rate of uninfested wheat stems ((relative to lodged stems on a log-odds scale on a log-odds scale) and mean yield per wheat head in the uninfected category for wheat variety (columns), seeding density (rows), and herbicide application rate. Arrows represent the median change on the vertical and horizontal axes with increasing *Bromus tectorum* pressure and contour lines are 95%, 75% and 50% highest posterior density region. Crop variety treatments are drought tolerant spring wheat (DT) and *Cephus cinctus* tolerant spring wheat (SFLT).

When the two pests were evaluated together, it was possible to detect a negative association between *C. cinctus* infestation of wheat and *B. tectorum* pressure (Fig. 4). These results indicated that *C. cinctus* appeared to prefer *B. tectorum* to wheat. This behavioral pattern resulted in areas of low *C. cinctus* infestation in wheat associated with high *B. tectorum* abundance. Our results also suggested that *B. tectorum* pressure interacted with wheat variety and seeding rate in determining *C. cinctus* oviposition behavior (Equation 2, Equation 3). An inspection of the impact of management practices on the joint posterior density of the proportion of uninfested wheat stems and mean yield per wheat head in the uninfested category indicated a complex response in *C. cinctus* oviposition behavior (Fig. 5). For five of the six tested crop varieties and seeding rate combinations and as postulated by our second oviposition behavior scenario, a decrease in herbicide rate, a proxy for increased *B. tectorum* pressure (Fig. 3), associated with lower mean wheat yield per head in the uninfested category. This result suggests that *C. cinctus* modified its oviposition behavior to select wheat stems of lower yielding potential, with cutting of *B. tectorum* by *C. cinctus* at about 1% of infested stems. Nevertheless, and in partial contradiction with the above postulated second oviposition scenario, the relative proportion of uninfested wheat stems increased with crop seeding rate. Specifically, at high crop densities, increasing *B. tectorum* pressure was associated with increased rates of uninfested wheat stems.

**Fig 6:**
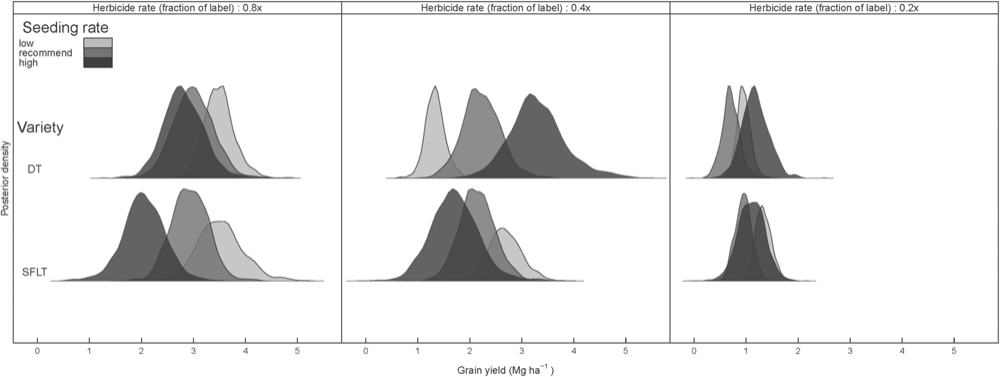
Posterior predicted yields for drought tolerant spring wheat (DT) and *Cephus cinctus* tolerant spring wheat (SFLT) spring wheat varieties at three seeding densities and under 0.8x, 0.4x, and 0.2x herbicide rate treatments (columns).

The impacts of *B. tectorum* on *C. cinctus* described above and the responses of these two pests to management practices were jointly modeled (Equation 1–3) and collected (Equation 4) to derive an estimate of crop yield that reflects the underlying process of multi-pest effects under contrasting management scenarios. This information, in turn, allowed the determination of the best management decisions given the most probable wheat yield outcome (Fig. 6). Results indicated that at low *B. tectorum* pressure (i.e. 0.8x herbicide rate) the low seeding rate SFLT variety had highest yields, 12.3% higher on average than DT at low seeding rates. However, the yield advantage of the SFLT variety decreased with increasing crop seeding rates to yields lower than the DT variety when they were planted at high seeding rates. A crop seeding rate by crop variety interaction became apparent at increased *B. tectorum* pressure. While yield remained negatively associated with higher seeding rates in SFLT wheat variety, increasing seeding rates of DT wheat variety had a positive effect on yield, probably due to its increased competitive ability against *B. tectorum*. Consequently, at intermediate *B. tectorum* pressure (i.e. 0.4x herbicide level), high seeded-DT was the highest yielding management decision, despite *C. cinctus* infestation. At intermediate *B. tectorum* pressure, the high-seeded SFLT resulted in the lowest yielding combination, perhaps a consequence of a lack of pith expression and increased *C. cinctus* pressure. At very high *B. tectorum* pressure (i.e. reduced herbicide rate of 0.2x), weed competition had a significant and overriding negative effect on crop yields with a slight advantage of higher seeding rates compared with low and recommended rates, particularly in the DT variety.

## Discussion and Conclusions

Current pest management strategies typically consider problems in isolation from each other, potentially leading to undesirable responses [2]. As such, non-integrative single-pest management approaches do not meet societal demands to develop holistic programs with reduced dependence of off-farm synthetic inputs. This study demonstrated that, even in the relatively low diversity agricultural system that dominates the Northern Great Plains, interactions between pest groups belonging to different trophic levels could complicate the outcome of otherwise seemingly simple management decisions. Our experiment, analysis, and results are consistent with applying a systems approach to modeling. Specifically, we developed hypotheses of causal relationships from key structural and functional aspects of the system which allowed us, in turn, to generalize relevant system dynamics and represent them in a simple manner consistent with management goals [41].

The observed pattern of reduced infestation of wheat in areas with high *B. tectorum* pressure agrees with previous observations that suggested *B. tectorum* as a preferred host for oviposition by *C. cinctus*, but with a very low level of successful larval development [19], [15], [40]. Thus, this study indicates that *B. tectorum* acts as a strong ecological sink for *C. cinctus*. Furthermore, the joint evaluation of *C. cinctus* infestation rates and grain yield reductions in the presence of *B. tectorum* revealed new insights into *C. cinctus* oviposition behavior. Specifically, our results indicated that at lower seeding rates and greater *B. tectorum* pressure, *C. cinctus* seemed to compromise offspring-performance [42] by ovipositing in significantly lower yielding stems and maintaining a relatively constant level of infestation in the crop. Future manipulative studies should quantify changes in oviposition behavior as a function of wheat and *B. tectorum* relative abundance to improve model precision.

An advantage in the Bayesian approach proposed here is that the effects of a particular management scheme on pest interactions and subsequent impact on crops was formally incorporated in the probabilistic estimates of yields. This capability becomes advantageous in the context of multi-pest species management because yield estimates reflected the effect that any single management decision had on each pest levels, their interactions, and subsequent joint impact. For example, at low *B. tectorum* pressure, our model predicted the low seeding rate of *C. cinctus* resistant wheat as the best management decision. At high seeding rates, the *C. cinctus* variety lost the insect-pest control advantage, probably due to lack of pith expression [43], and the drought tolerant variety planted at high seeding rates became the best management decision, regardless of *C. cinctus* abundance. In accordance, in the absence of *B. tectorum*, Beres et al. [44] found that solid stem wheat sown at 150 seeds m^-2^ had higher but inconsistent pith expression compared with densities of 250–450 seeds m^-2^, suggesting a negative effect of interspecific competition on pith expression and *C. cinctus* control.

The joint evaluation of insect pests, weeds, and crop yield is essential given the existence of multitrophic interactions occurring within agroecosystems, but this is often conducted for each measure separately [45], [46]. Under this standard approach, results of statistical inference only describe the current state of pest levels and crop yields. To inform management decisions, statistical analysis must be qualitatively assessed under realistic scenarios that consider all potential combinations of pest levels. Our approach allowed us to jointly model estimates of *B. tectorum* and *C. cinctus* pressure, wheat stem counts, and grain yield, formally quantify how their interactions are modified by management decisions, and assess their yield implications. This approach provided insights that a standard model would not have revealed. In doing so, our work exemplifies the necessity of accounting for direct and indirect interactions between multiple pests in the agroecosystem, and propagating this information from the statistical analysis stage to the model used to provide management decision.

In recent years, increased input costs, non-target impacts, and pesticide resistance issues associated with single-species pest management programs had compromised the robustness, resilience, and environmental integration required in sustainable farming systems [47]. For example, the continued expansion and intensification of the small grain production system has resulted in the selection of several herbicide resistant weed biotypes, including cases of multiple herbicide resistance [48]. While our study evaluated approaches to model the joint impact of management decision and multitrophic interactions on crop outputs, it also allow us contemplate alternative applied scenarios. For example, minimizing the selective pressure of herbicide resistance may necessitate making choices as to when and where it is most sustainable to utilize a weed-competitive wheat variety, an approach that could allow *C. cinctus* numbers to increase. This approach will reduce frequency of herbicide applications, reducing the selective pressure towards herbicide resistance. When needed, herbicide applications should be done in conjunction with the use of *C. cinctus* resistant solid stem varieties to allow the decline in insect numbers while also maximizing the weed control that has been inhibited by the most competitive wheat variety over the previous years. If successful, this could be achieved within a single season, before returning to that herbicide practice for several more years.

The search for alternative row-crop agricultural systems that attenuate, avoid, or even reverse the environmental and biological harms associated with conventional practices has resulted in an increased interest in developing alternative management systems. Doing so requires a system-level understanding of the ecological and management processes that underpin biological interactions occurring within crop fields [49]. Hopefully, the outcomes from this innovative modeling strategy will help to further advance the development of holistic decision-aid tools in the integrated management of agricultural pests.
